# Social attention and social-emotional modulation of attention in Angelman syndrome: an eye-tracking study

**DOI:** 10.1038/s41598-023-30199-6

**Published:** 2023-02-28

**Authors:** Serena Micheletti, Giacomo Vivanti, Stefano Renzetti, Matteo Paolo Lanaro, Paola Martelli, Stefano Calza, Social Attention in Angelman Study Group, Patrizia Accorsi, Stefania Agostini, Anna Alessandrini, Nicole D’Adda, Laura Ferrari, Valentina Foresti, Jessica Galli, Lucio Giordano, Melissa Marras, Alessandro Rizzi, Elisa Fazzi

**Affiliations:** 1 Unit of Child Neurology and Psychiatry, ASST Spedali Civili of Brescia, Piazzale Spedali Civili 1, 25123 Brescia, Italy; 2 AJ Drexel Autism Institute, Drexel University, Philadelphia, PA USA; 3 Department of Medical-Surgical Specialties, Radiological Sciences and Public Health, University of Brescia, Brescia, Italy; 4 Department of Computer Science, University of Milan, Milan, Italy; 5 Unit of Biostatistics and Bioinformatics, Department of Molecular and Translational Medicine, University of Brescia, Brescia, Italy; 6 Department of Clinical and Experimental Sciences, University of Brescia, Brescia, Italy

**Keywords:** Behavioural genetics, Human behaviour, Social behaviour

## Abstract

Individuals with Angelman syndrome (AS) present with severe intellectual disability alongside a social phenotype characterised by social communication difficulties and an increased drive for social engagement. As the social phenotype in this condition is poorly understood, we examined patterns of social attention and social modulation of attention in AS. Twenty-four individuals with AS and twenty-one young children with similar mental age were shown videos featuring unfamiliar actors who performed simple actions across two conditions: a playful condition, in which the actor showed positive facial emotions, and a neutral condition, in which the actor showed a neutral facial expression. During the passive observation of the videos, participants’ proportion of time spent watching the two areas of interest (faces and actions) was examined using eye-tracking technology. We found that the playful condition elicited increased proportion of fixations duration to the actor’s face compared to the neutral condition similarly across groups. Additionally, the proportion of fixations duration to the action area was similar across groups in the two conditions. However, children with AS looked towards the actor’s face for a shorter duration compared to the comparison group across conditions. This pattern of similarities and differences provides novel insight on the complex social phenotype of children with AS.

## Introduction

Angelman syndrome (AS) is a rare and disabling neurodevelopment disorder (estimated incidence 1 in 12.000—20.000^1^) caused by a disruption of the maternally-inherited UBE3A gene^2^. Expressive language delay and difficulties in joint attention and dyadic engagement are key features in children with AS, who also manifest severe intellectual disability, epilepsy, ataxia and sleep disorders^1,3^.

Additionally, the AS phenotype is paradoxically characterised by both social communication difficulties and an increased drive for social engagement. Indeed, individuals with AS display an atypically increased frequency of smiling and laughing, particularly in highly-engaging social settings^4^. Nevertheless, they exhibit challenging behaviours, that include—but are not limited to—restlessness, hyperactivity, lack of compliance, aggressive behaviour^5^, temper tantrums, and severe inattentiveness^6^. These maladaptive behaviours interfere with their ability to interact in social environments.

Recent research has shown a distinctive profile of imitation – an important dimension of social functioning – in children with AS, characterised by increased imitation in response to models showing an “emotionally playful” affect, compared to models displaying a neutral emotional expression^7^. Although in the same study typically developing children matched by developmental age were also showing enhanced imitation in response to emotional versus neutral models, the magnitude of the enhancement was higher in participants with AS. This unique AS social phenotype has the potential to provide insight into the mechanisms underlying social communication in typical and atypical development.

Insight into these phenomena might be gained through the eye-tracking technology, which is an infra-red camera system that uses the contrast between the pupil and the iris to locate the centre of the pupil and infrared light to create a corneal reflection. The vector between these two features is then used to compute gaze intersection with a surface, thus measuring the focus of attention or where a person is looking. The measurement of what individuals attend to can reveal important information on how stimuli are prioritised, experienced, and processed, allowing researchers to “look through the eyes” of their research participants. Due to its non-invasive nature and limited requirement for test-taking skills, eye-tracking technology has been used to study attentional patterns in different neurodevelopmental disorders^8,9^, particularly in Autism Spectrum Disorder (ASD), providing insights relevant to subgroup differentiation, symptomatology definition and understanding of mechanisms underlying clinical manifestations^10–12^. Through this technology, important information regarding attention towards social stimuli can be studied as well^12,13^.

The application of this technology to subjects with AS can be challenging due to motor control and motor coordination difficulties^3^, limited attention span^14–16^ and neuro-visual dysfunctions (astigmatism, reduced visual acuity and oculomotor dysfunctions) which can affect the ability to orient to stimuli. However, recent work by Hong et al.^17^ has pointed to the viability of eye-tracking technology in the study of social attention in AS. Specifically, Hong et al.^17^ compared eye-movements among individuals with AS, ASD and typically developing controls in response to social scenes or geometric patterns presented side by side in a simultaneous fashion. Results of this study suggested that less than half of individuals with AS attended to the social scenes and geometric patterns, and, among those who completed the task, AS participants looked at social scenes consistently less than to geometric shapes compared to typically-developing children. Surprisingly, this attentional pattern was similar to that of participants with ASD. These results are puzzling in the context of the enhanced drive for social engagement in AS (in contrast to ASD) and point to the need for further research on how social-emotional cues modulate attention and behaviour in AS.

The present study was designed to expand on the findings by Micheletti et al.^7^, with the goal of examining social attention and the social-emotional modulation of attention in individuals with AS. The eye-tracking technology was used to examine attentional patterns, in terms of fixations duration towards video-stimuli depicting a person performing an action displayed on a computer screen, in children with AS and age-matched typically-developing children, with the goal of addressing the following questions:

- (1)Do individuals with AS differ from mental age—matched children with typical development, in terms of proportion of time spent watching the two areas of interest (faces and actions) out of their total fixations duration to the screen? Based on the results of Micheletti et al.^7^ and previous literature on enhanced social drive in AS, we predicted that (a) both groups would display higher proportion of fixations duration (P-FD) towards the actor’s faces compared to her actions, and (b) the increased P-FD to the actor’s face versus her action would be significantly higher in participants with AS.
- (2)In participants with AS, is the P-FD towards faces and actions influenced by whether the actor in the video shows an emotionally engaging versus neutral affect? We predicted that (a) both groups would show an increased P-FD to the actor’s face in response to actors showing emotionally engaging versus neutral affect, and (b) the increased P-FD towards the actor’s face in the playful versus neutral condition would be significantly more pronounced in the ASG.
- (3) Additionally, we explored whether the time spent watching videos was associated with chronological and mental age in AS and in the mental age—matched children.

## Results

Among the 24 participants with AS who were enrolled in the study, 4 did not complete the evaluation protocol because of severe difficulties in the eye-tracker calibration phase (a female and a male with UBE3A deletion) or severe difficulties in maintaining the sitting position while watching videos (2 females with UBE3A deletion).

All the children in the control group (CG) completed the experiment. Among those who completed the evaluation, 3 participants in the ASG and 2 in the CG appeared to be excessively distracted and needed to take a break before continuing with the experiment.

**Figure 1:**
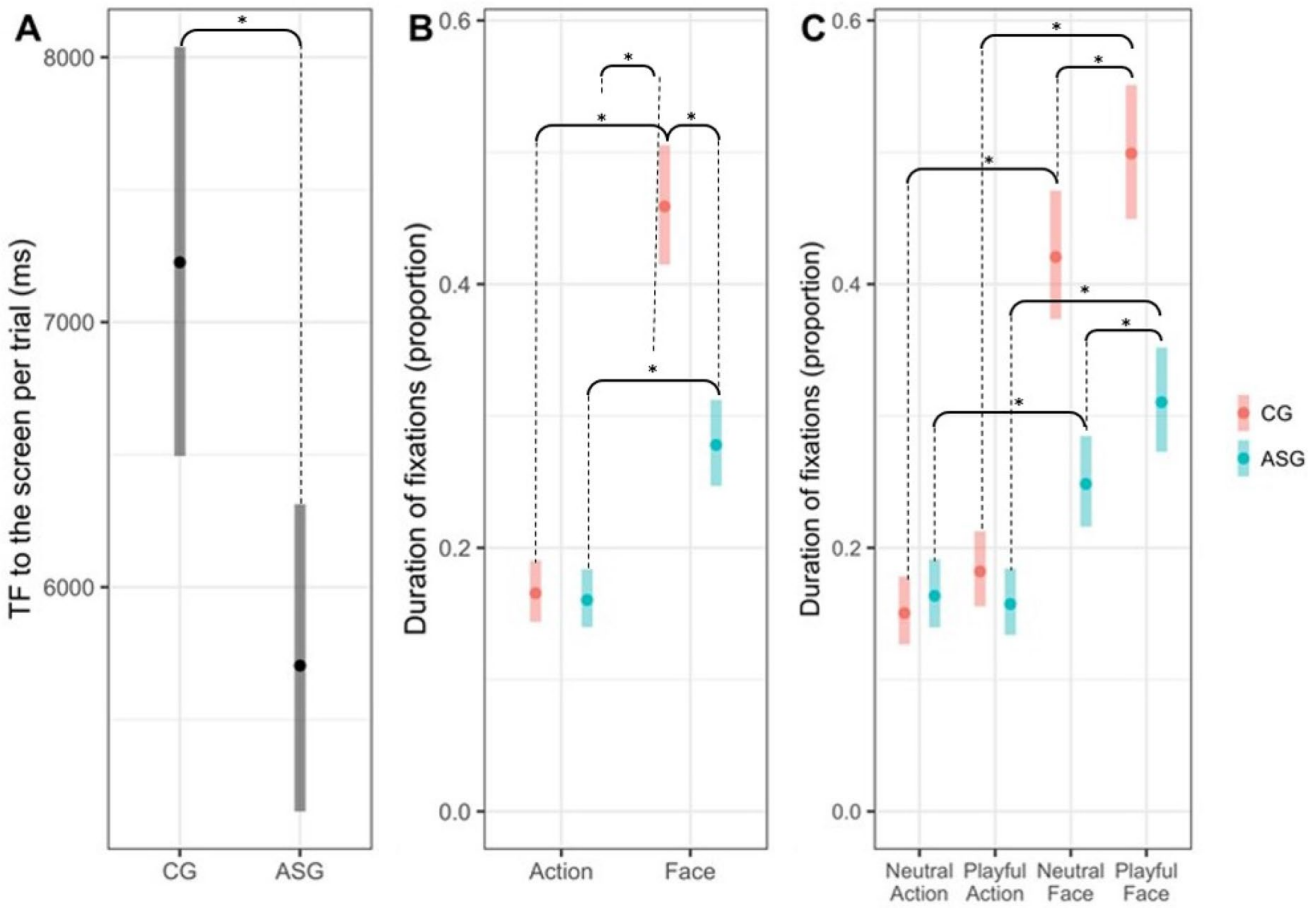
Results from the generalised linear mixed effect models (*= p < 0.05). Panel (**A**) shows the Total Fixations Duration (TF) per trial in CG and ASG estimated through a negative binomial mixed effect model. Panel (**B**) represents the proportion of fixations duration (P-FD) to the areas of interest (face, actions) in CG and ASG. Panel (**C**) represents the P-FD to the areas of interest across conditions (playful and neutral) in CG and ASG. Both results in panel B and C were obtained from a beta mixed effect regression model. Bars represent the 95% confidence intervals estimated by the regression models.

We first conducted preliminary analyses testing whether the groups differed in terms of total fixations duration towards the screen (TF) per trial using all the videos across conditions combined and found that the ASG looked at the screen area less time compared to CG (CG/ASG = 1.267; 95% CI 1.093, 1.468; p = 0.002- Fig. 1A, Table 1S)*.* We also tested whether gender was associated with TF and found no significant difference across the CG (p = 0.698) and ASG (p = 0.362) between males and females.

Moreover, considering the two different conditions (playful versus neutral videos), the ASG spent significantly less time watching the screen compared to the CG, regardless of the level of social engagement expressed by the actor (TF playful condition CG-ASG = 0.088; 95% CI 0.028, 0.148; p < 0.001; TF neutral condition CG-ASG = 0.278; 95% CI 0.001, 0.110; p = 0.042).

We subsequently addressed our research question related to the P-FD shown by the two groups towards the two areas of interest (faces and actions). Consistent with our first prediction, the face area elicited higher P-FD compared to the action area across participants, as indicated by a main effect of Area of Interest (face, action p < 0.001; ASG Action—ASG Face = − 0.106; 95% CI − 0.145, − 0.068; p < 0.001; CG Action—CG Face = − 0.287; 95% CI − 0.347, − 0.227; p < 0.001). Additionally, there was a main effect of Group, indicating that P-FD was lower in the ASG across areas of interest (p < 0.001). Additionally, contrary to our hypotheses, a significant Group (ASG, CG) X Areas of Interest (face, action) interaction effect was observed (p < 0.001), with CG focusing their fixations proportionally more towards faces compared to actions, compared to ASG. In fact, while the P-FD to the action area was similar across groups (CG-ASG = 0.031; 95% CI − 0.017, 0.078; p = 0.354), the ASG showed a lower P-FD to the face area compared to CG (CG-ASG = 0.212; 95% CI 0.122, 0.302; p < 0.001, Fig. 1B)*.* Therefore, our first prediction (across groups the face area would elicit higher P-FD than the action area) was supported, while our second prediction (the increased P-FD to the actor’s face versus her action would be significantly more pronounced in participants with AS) was not supported, with data pointing to the opposite pattern.

We then examined the P-FD towards the two areas of interest (the actors’ face and action), in response to the different conditions (playful versus neutral) across groups (Fig. 1C). A beta mixed effect model was conducted.

With regards to the P-FD towards faces, a post-hoc analysis on simple main effects from the Area of Interest X Condition interaction revealed a main effect of Condition (playful versus neutral, p < 0.001), indicating that overall the playful condition elicited increased P-FD towards the actors’ face across participants. The same analysis was performed considering the simple main effects from the Area of Interest X Group interaction. A main effect of Group (ASG, CG) was detected, indicating decreased P-FD to the actors’ face across the playful and neutral conditions in the ASG compared to the CG (neutral face: CG-ASG = 0.182; 95% CI 0.066, 0.298; p < 0.001; playful face: CG-ASG = 0.242; 95% CI 0.115, 0.370; p < 0.001). Additionally, there was no Group (AGS, CG) X Condition (playful vs. neutral) interaction effect (p = 0.785).

With regards to the P-FD towards actions, we examined the simple main effects of the Area of Interest X Condition interaction in response to the playful versus neutral condition. Results showed no main effect of Condition (playful versus neutral, p = 0.201), indicating that the playful condition did not elicit increased P-FD towards the actors’ actions across participants. Additionally, there was no main effect of Group (ASG vs. CG) when considering the simple main effects from the Area of Interest X Group interaction, indicating similar P-FD to the actors’ actions across the playful and neutral conditions across groups (p = 0.748; neutral action: CG-ASG = 0.007; 95% CI − 0.058, 0.071; p = 1.000; playful action: CG-ASG = 0.056; 95% CI − 0.013, 0.125; p = 0.208). Additionally, there was a non-significant trend towards a Group (AGS and CG) X Condition (playful vs. neutral) interaction effect (p = 0.053) suggesting that the P-FD towards actions was differentially affected by the two conditions in the two groups, with ASG group slightly more interested in fixating neutral actions than playful ones, while the opposite pattern appeared to be shown by the CG. However, follow-up analyses indicated that this trend did not reflect statically significant differences in the P-FD towards the action area across groups (Action neutral – playful = 0.011; 95% CI − 0.033, 0.056; p = 0.994, CG Action neutral – playful = − 0.038; 95% CI − 0.094, 0.018; p = 0.431).

Finally, in each condition (playful and neutral), a Group (ASG, CG) X Areas of Interest (face, action) interaction effect was found with participants showing higher P-FD towards the face area compared to the action area in each condition (ASG Neutral action – ASG Neutral face = − 0.076; 95% CI − 0.134, − 0.018; p = 0.002; ASG Playful action – ASG Playful face = − 0.139; 95% CI − 0.204, − 0.074; p < 0.001; CG Neutral Action—CG Neutral Face = − 0.251; 95% CI − 0.339, − 0.163; p < 0.001; CG Playful Action—CG Playful Face = − 0.325; 95% CI − 0.418, − 0.233; p < 0.001).

This pattern of findings indicates support for our prediction that the emotionally engaging condition would elicit higher P-FD to the actor’s face compared to the neutral condition across groups. Conversely, our second prediction (that increased P-FD to the actor’s face in the playful versus neutral condition would be significantly higher in the ASG) was not supported.

**Figure 2:**
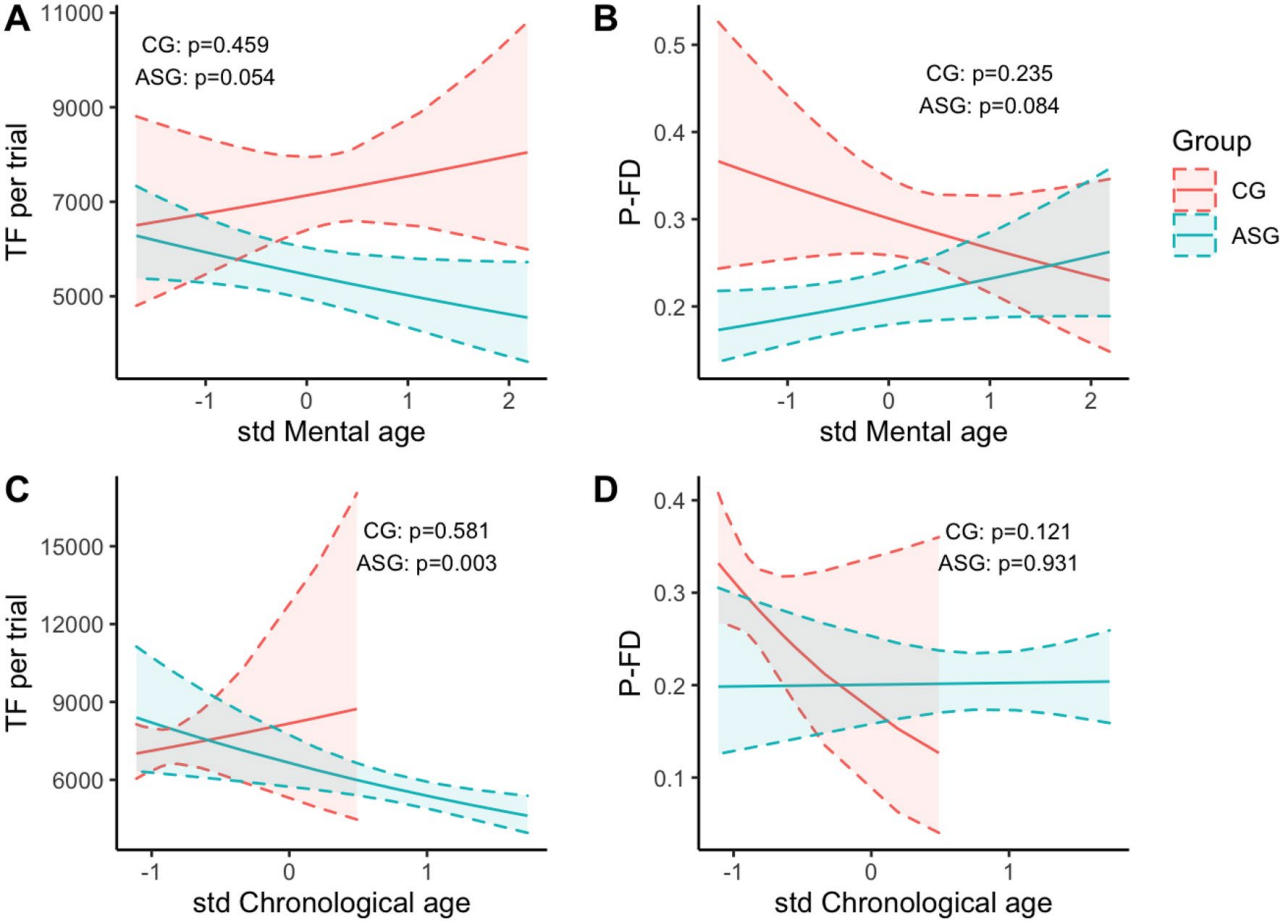
Results from the mixed effect models obtained to test for the association between mental age and TF—total fixations duration—(**A**) and P-FD—proportion of fixation duration—(**B**) and between chronological age and TF (**C**) and P-FD (**D**). TF is expressed in milliseconds and is considered per trial. Chronological and mental age were standardized. The dashed lines represents the 95% confidence intervals estimated by the regression models.

Finally, to address our third research question, we explored whether participants’ chronological and mental age was associated with the time spent watching the screen (TF) and the areas of interest in the two groups (Fig. 2). In CG, TF was not related to the increased chronological and mental age (respectively p = 0.459 and p = 0.581). On the contrary, AS individuals with higher mental and chronological age showed lower TF (respectively p = 0.054 and p = 0.003). No significant association was found between the time spent watching the areas of interest and mental/chronological age in either group (mental age, ASG: p = 0.324, CG: p = 0.298; chronological age, ASG: p = 0.263, CG: p = 0.153). As shown in Fig. 2, the interaction terms between mental or chronological age and the grouping variable were not statistically significant across the four models (TF vs. mental age, p = 0.108; TF vs. chronological age, p = 0.177; P-FD to the interest area vs. mental age, p = 0.058, P-FD vs. chronological age, p = 0.129) meaning that there was no statistically significant differences between the two groups. Overall TF (without group distinction) was negatively associated with chronological age (p = 0.007) while P-FD to the specific areas of interest was not (p = 0.742).

## Discussion

Previous research has shown that individuals with AS display an increased drive for social engagement, and imitate adults showing an “emotionally playful” attitude more often compared to “neutral” adults. In our previous study this modulation was more pronounced compared to typically-developing children^7^.

Against this background, the present study examined whether children with AS show increased social attention, as indicated by a higher proportion of fixations duration towards faces versus actions, and an increased social-emotional responsivity, expressed by the total fixations duration and proportion of fixations duration to emotionally engaging stimuli versus neutral ones. We found a mixed pattern of results, with limited support for our initial hypotheses. Although, as predicted, the face of the actors in video-stimuli elicited higher proportion of fixations duration than her actions across conditions in both groups, our hypothesis that the increased proportion of fixations duration to the actor’s face versus her actions would be more pronounced in participants with AS was not supported. Indeed, contrary to our hypotheses, the increased proportion of fixations duration to faces versus actions was more pronounced in the CG compared to the ASG, and overall participants with AS proportionally watched the face of the actor depicted in the video-stimuli to a lesser degree compared to mental age-matched typical participants.

Additionally, although our prediction that the emotionally engaging condition would elicit higher proportion of fixations duration towards the actor’s face compared to the neutral condition across groups was supported, we found no evidence supporting our prediction that the increased proportion of fixations duration towards the actor’s face in the playful versus neutral condition would be more pronounced in the ASG. Another relevant finding is that participants in the ASG spent significantly less time watching the screen compared to the CG. This pattern of results suggests that, in the context of an overall reduced attentional engagement with the stimuli presented to them, children with AS increase their proportion of fixations duration in response to social-emotional signals (the playfulness expressed by the actor’s face) but not more than children in the comparison group. Additionally, their tendency to increase their proportion of fixations duration in response to faces versus actions appeared to be lower than in neuro-typical children. Taken together, these results are inconsistent with the notion of hyper-responsivity to social and emotional stimuli in children with AS upon which our hypotheses were developed.

Importantly, however our finding that individuals with AS showed a lower proportion of fixations duration to the actor’s face compared to participants in the CG is consistent with the only previous study that used eye-tracking in subjects with AS^17^. The lower proportion of fixations duration towards face stimuli presented on a screen compared to mental age-matched controls is unlikely to be explained by general intellectual reasoning deficits in AS, as in both studies a reduced fixations duration was documented in comparison to participants with a similar mental age. Another possibility is that group differences observed in our findings reflect a general attentional problem in AS (e.g. increased distractibility). However, the proportion of fixations duration towards the action performed by the actor on the screen was similar across groups, suggesting that general attentional deficits, while present, might not be a satisfying explanation for the differential proportion of fixations duration to the face area. It is therefore plausible that the reduced visual engagement with the actor’s face documented in the ASG reflects a distinctive clinical manifestation related to the social domain that co-exists with a general inattentiveness, rather than reflecting a mere byproduct of intellectual disability or inattentiveness/distractibility. Reduced social attention might also be related to reduced social reasoning in AS by virtue of reflecting an abnormally heightened arousal in response to social stimuli, which might lead to both increased social approach and social processing difficulties, as observed in Williams syndrome^13^.

Additionally, we found that, despite an overall reduced total fixations duration to the screen, children with AS modulate their attention in response to the emotional signals expressed by the actors in the video. These sensitivity and responsiveness to social-emotional signals are consistent with the clinical presentation of AS. Indeed, individuals with AS are known to have prominent interest in social interaction^18^; they laugh and smile with a high frequency with familiar people, when eye contact is maintained^19^ and when it is elicited by social environmental stimuli^20^; additionally they show responsivity to social-communicative cues, imitating adults who act in a socially engaging manner versus a neutral one^7^.

Finally, the current results show that, counterintuitively, children in the ASG who had longer fixations duration to the screen across conditions and areas of interest had lower mental and chronological age. This unexpected negative correlation between the mental/chronological age and total duration of fixations in AS could be due to the child-oriented video content being of less interest to the older participants (i.e. those with more social experience could have found the depicted actions not as engaging as the younger children). A negative correlation between age and social engagement, expressed in a reduced amount of time watching different pictures representing happy faces, has been previously found in children with Rett syndrome^21^, and attributed to the consequences of their non-verbal and limited communication abilities reducing opportunities for social experiences and affecting social interest. Adams et al.^4^ showed age-related changes in the sociability of children with AS, reporting not only a decrease in the duration of laughing and smiling as the children get older, but also reduced responsivity to social interaction and eye contact. This age-related decline in social motivation and responsivity may contribute to the counterintuitive negative correlation between chronological/mental age and attention to the stimuli in our study, but further research is needed to substantiate this explanation.

Several implications can be drawn from the study findings. First, the findings are aligned with the literature documenting that typically developing children increase their attentional engagement when viewing stimuli that are more socially salient (faces as opposed to actions) and more emotionally salient (playful versus neutral actions). This phenomenon is considered to play a critical role in the development of social communication, as an increased engagement with social and emotional stimuli allows children to register and respond to important social cues like gaze direction, affect, as well as other verbal and nonverbal information. The prioritization of frequent engagement with these stimuli over non-social and non-emotional stimuli, in turn, is believed to shape neural specialization and behavioral expertise in the social domain during sensitive periods^22–24^.

Importantly, our study found that children with AS also attend more to social versus non-social and emotional versus neutral stimuli – however, this modulation is not more pronounced in this group as we predicted. Indeed, the unusual social phenotype in AS, characterized by both social processing impairments (e.g. difficulties in joint attention) and increased motivation for social approach, led us to hypothesize that children with this condition are hyper-responsive to social and emotional stimuli. This notion could provide a potential explanation for both processing difficulties, as a hyper-response to social stimuli could affect cognitive processing in the social domain while enhancing social motivation. However, our results are not consistent with this explanation, pointing to the need for different explanatory frameworks for the complex social phenotype of those with AS. Importantly, our finding that children with AS preferentially attend social and emotional stimuli points to the potential for interventions to capitalize on social attention and social-emotional modulation to facilitate early learning. Several intervention practices developed for other neurodevelopmental conditions are designed to increase the saliency of social signals (e.g. through exaggerated facial and affective cues) to enhance attentional engagement and processing of others’ communication and actions^25^. Recent research has shown that these techniques can facilitate early learning across a variety of domains, including areas that are affected in AS, such as joint attention, imitation and adaptive social behaviors^26^. Our results provide a rationale for extending such interventions to children with AS.

Our study has several limitations that should be acknowledged. First, the stimuli presented to participants were displayed through a computer screen, providing an experience that might be processed differently compared to real-life interactions. This could have disproportionally penalized participants in ASG, as AS has been associated with neuro-visual dysfunctions affecting the processing of complex visual scenes^27–29^, difficulties in recognising facial expressions and difficulties processing “impoverished” visual information, such as the 2-D information presented via the computer screen^30,31^. Although these visual-attentional difficulties could have contributed to our findings, the ASG had proportion of fixations duration towards the action area similar to CG and normative emotional modulation, suggesting that our paradigm was able to capture social-domain specific patterns. However, a live version of the paradigm would have allowed ascertaining whether group differences documented here reflected a true reduction in social attention versus difficulties specific to processing 2-D social information^30,31^. Such a paradigm could be coupled with the use of eye-tracking glasses to maximise both ecological validity and measurement precision.

Another limitation is the wide range in chronological age in the ASG, due to the recruitment difficulties associated with the low prevalence of this syndrome. Additionally, the groups were not gender-matched. However, no gender effect was found across our variables of interest.

One additional limitation is the use of a comparison group matched by mental age but not chronological age. Although this strategy was designed to ensure that participants had similar general intellectual reasoning abilities across groups, future research should attempt to replicate our results using alternative matching strategies, including both chronological and mental age-matched comparison groups. Importantly, the chronological age difference between the groups could have affected results due to the different histories of social experiences in the two groups. However, although participants in the ASG had theoretically a longer history of social interactions than control participants by virtue of being older, cognitive impairments associated with AS likely limited the breadth and range of social experiences – consistent with our finding that older children in the ASG did not show more social attention and other literature with other syndromic conditions and similar intellectual profile^21^. Furthermore, several factors that were not controlled for in the analyses could have affected participants’ performance. These include the gender (female) of the actors in the videos (although research suggests that males and females are attended to a similar amount of time in children in the age range of participants in the study^32,33^), as well as treatments and interventions received by participants in the AS group. For example, specific trainings addressing maladaptive behaviors in the social domain (e.g. aggression, inappropriate social approach) could have affected results directly (contributing to attentional social engagement with the stimuli) or indirectly (by affecting compliance with instructions). An additional limitation is that the study did not assess how eye-tracking patterns related to clinical dimensions of AS other than mental age, including maladaptive behaviors, thus limiting the implication that can be derived from the results. Future research should examine the potential role of these and other factors that were not controlled for or measured in the current study, and also focus on a more fine-grained analysis of moment-by-moment changes in the position of fixations to examine to gain more insight into how events displayed in the videos affect gaze patterns in this population.

Despite these limitations, to our knowledge, this is the first controlled study focusing on social attention and social modulation of attention in individuals with AS, thus providing new insights into the social phenotype of this under-studied population.

In conclusion, our study documented both typical social processes (increased proportion of fixations duration towards social-emotional versus neutral stimuli) and atypical ones (diminished proportion of fixations duration towards faces) in individuals with AS, and did not support the notion of enhanced social attention or social modulation in this population. Further research is needed to substantiate these results and to examine domain-general and social domain-specific mechanisms underlying these puzzling phenomena.

## Methods

### Participants

Twenty-four participants with AS (16 females, 8 males, mean chronological age of 11.8 years, SD = 9.1 years; age range = 2.7 years–33.7 years), were recruited either via our children hospital unit or through the Italian Angelman Syndrome Organisation (OR.S.A.) during their annual conference.

The inclusion criteria for the ASG consisted in a molecular confirmed diagnosis of AS, well-controlled epileptic seizures, a visual acuity of more than 3 dec., primary home-spoken language of Italian (L1 Italian learners), lack of uncorrected visual or hearing impairments and a lack of further severe medical conditions beyond AS. In terms of genetic profiling, 16 individuals in the ASG presented a UBE3A deletion, 5 presented a UBE3A mutation and the remaining 3 a uniparental disomy. Additionally, 18 individuals suffered from epilepsy and 14 of them were receiving antiepileptic therapies, with well-controlled seizures. Their mental age was measured using Griffiths Mental Developmental Scales – Extended Revised (Griffiths ER^34,35^, and was observed to be 20.1 months’ average (SD = 8.7 months, range = 9 months–38 months).

Their socialisation skills were tested through the Socialisation subscale of the Vineland Adaptive Behaviour Scales^36^, with an average value of 58.9 raw scores (SD = 14.1; range = 41–84 raw scores) and age equivalent scores that ranged from under 18 months to 28 months. All individuals in the ASG attended, or were attending at the time of the study, a school within the Italian mainstream educational system, supported by special education teachers. All of them lived at home with their parents and were familiar with watching digital videos, that were played daily, on a television, computer, mobile or tablet screen.

The CG included 21 participants (6 females, 15 males, mean chronological age: 23 months, SD = 5.23 months; age range = 18–35 months), with a similar mental age to the ASG (p = 0.328). Individuals in the CG were recruited among the patients normally referring to our children hospital with the purpose to attend orthopedic, paediatric, or surgical visits. Inclusion criteria were the absence of known medical conditions, within normal-range psychomotor development (Griffiths ER^34,35^ Developmental Quotient equal or higher than -1 standard deviation), typical language development, normal vision and hearing functions. An interview covering pre-peri-post natal events and psychomotor development anamnestic data was administered to those families agreeing for their child to participate in the study. This included social, motor and language development assessments. The children who satisfied the inclusion criteria were then administered the Griffiths ER^34,35^. Those who reached a standard score within the normal range were then included in the study and took part in the experimental task. No participant in CG was receiving medical therapies at the time of the study.

### Procedures

The current study was approved by the institutional review board of *ASST Spedali Civili of Brescia* (*Comitato Etico di Brescia*, ID number: ASET-NP 2890). All study procedures were performed following the relevant guidelines and regulations. Informed consent was obtained from all participants’ parents or legal guardians. Informed consent was also obtained in order to publish identifying images in an online open-access publication.

Participants attended two sessions which took place inside a quiet room of our Unit. The initial visit consisted in the administration of the Griffiths ER. Subsequently, participants meeting inclusion criteria were invited to attend the second session, which was held within 15 days from the initial visit and comprised the administration of the experimental eye-tracking paradigm. The eye-tracking paradigm was implemented via an EyeLink 1000 eye tracker connected to an LCD Monitor 23.6" (HP V241A). The computer ran SR Research Experiment Builder software (Version 2.1.512), which was adopted to display the stimuli and to record visual attention data expressed by the fixations duration. Data were analysed via SR Research EyeLink Data Viewer Software (Version 3.2.48).

**Figure 3:**
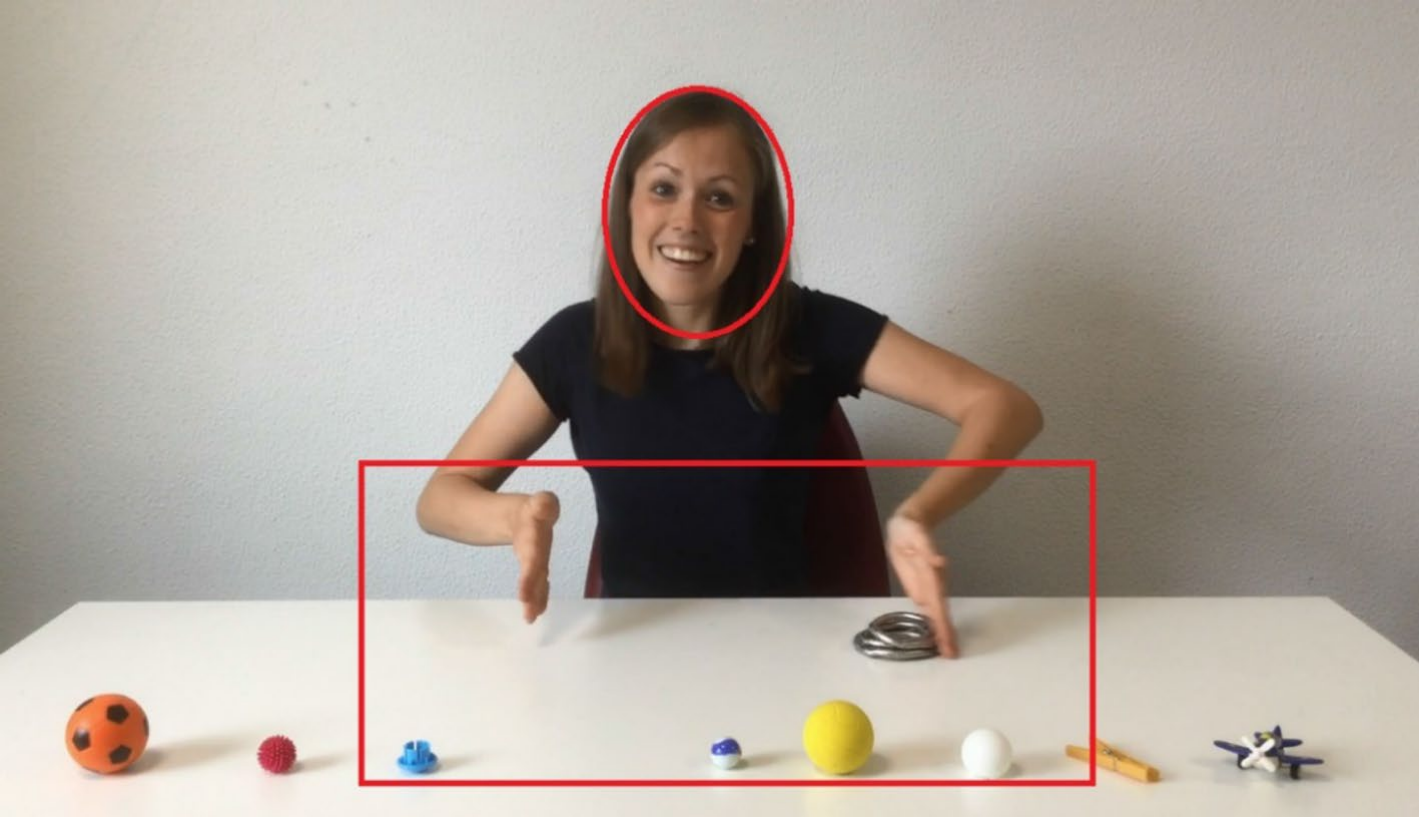
Video-recorded condition. The red oval line represents an interest area for face, the red rectangle line represents an interest area for action.

A calibration procedure was administered prior to testing each participant. The areas of interest for each stimulus were set for the head and the action performed by the actor, which included the actor’s hands and the objects being manipulated, as illustrated in Fig. 3.

**Figure 4:**
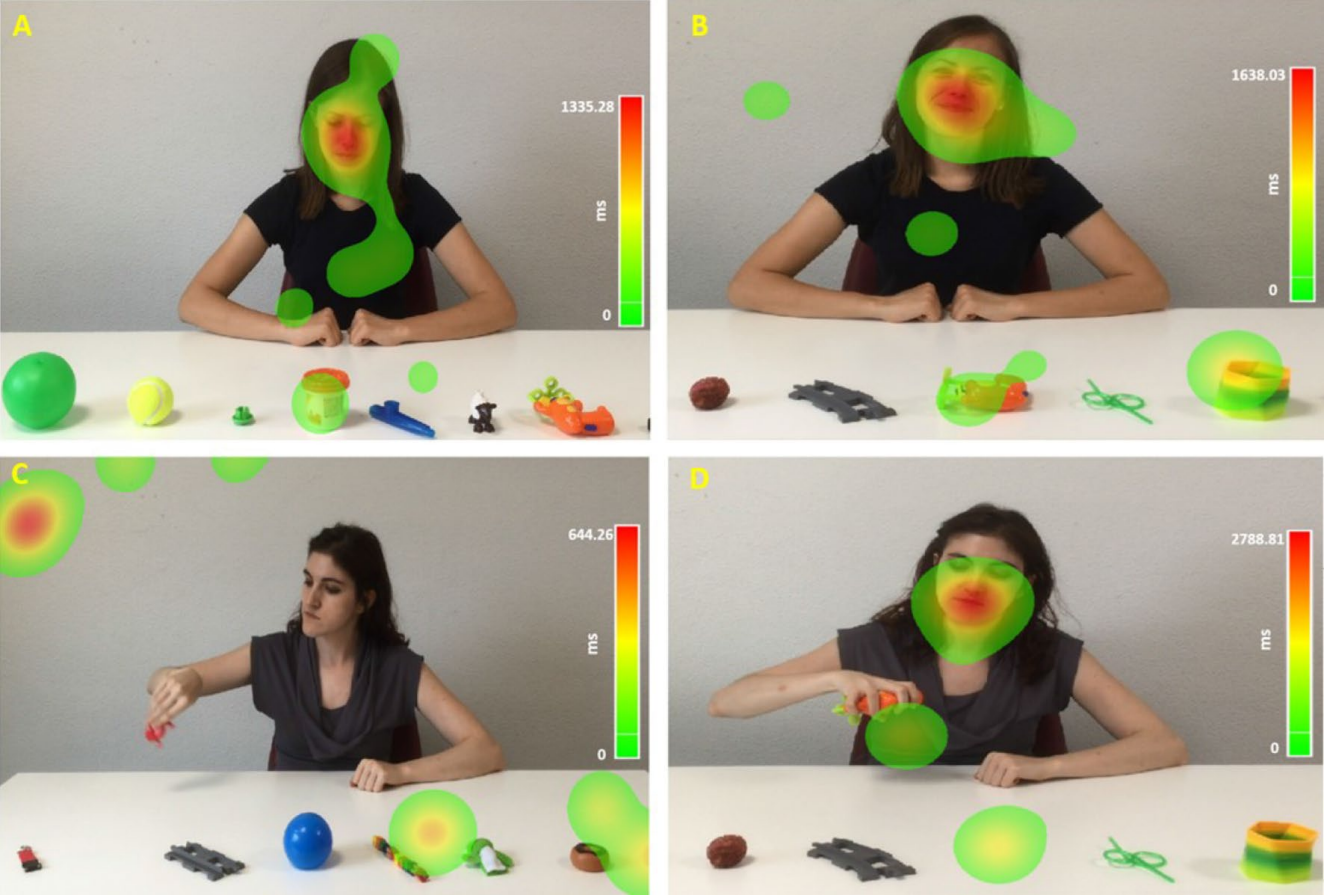
Examples of video-recorded playful (**A,B**) and neutral (**C,D**) conditions. Heat maps represent the time spent (ms) watching the video. Colors, from green to red, represent the increasing time spent watching the videos. In figures (**A–C**) a participant belonging to CG spent time watching both faces and action areas. In figure C the same participant is interested in objects and the screen, avoiding interest areas.

The experimental paradigm involved the passive viewing of 8 video stimuli (7 s each), randomly ordered. Participants were seated on a comfortable chair, facing the eye-tracker monitor positioned 50 cm from their heads. Each video depicted a sitting female actor performing a single simple action that involved one of the 9 objects placed in front of her on a white table. The actor alternated her gaze between the manipulated object and the participant, and her actions were always arbitrary in nature (e.g. placing a cone on the palm of the hand). There were two conditions; a playful and a neutral condition, which varied according to the positive or neutral emotional facial expression displayed by the actor (Fig. 4). In the playful condition, the actor performed the key action in a socially engaging manner, incorporating positive-affect emotional expressions as well as an animated body language. Comparatively, in the neutral condition another actor performed the same action, using a different object, while conveying a “neutral” facial and bodily emotional display. Video stimuli were arranged within the same fixed random order across participants in both groups. Each video displayed a novel action involving a different object from the set, ensuring no action/object pair was ever repeated in order to avoid confounding learning effects. The amount of time between the presentation of each video depended on the level of compliance of the individual. If the participant appeared to be excessively tired or distracted, the experiment was discontinued and a break was offered. Typical behaviours during these breaks were relaxing on the chair or walking around the room. After each break the eye-tracker would be recalibrated before proceeding with the next video presentation. We examined participants’ total fixations duration to the actor’s face and her actions and computed the proportion of time spent watching the two areas of interest out of their total fixations duration to the screen (proportion of fixations duration – hereafter P-FD—expressed in milliseconds). Participants in both groups required about 60 min to complete the first section of developmental quotient evaluation (Griffiths ER scales) and about 10 min for the second section.

### Statistical analysis

Generalised linear mixed-effect models were considered in the analysis to account for the repeated measure of each test. A negative binomial regression was applied when attention (expressed by fixations duration -TF) to the overall screen was the outcome while a beta regression was used when proportion of fixations duration to the areas of interest (face, actions) was the dependent variable. Comparisons of TF per trial between gender by group were tested through a Poisson mixed effect model. An interaction term among group, condition and area of interest was included in the model to test our hypotheses. Poisson mixed effect model was used to test for the association between mental or chronological age and TF, since there were counting variables, while beta mixed effect models were applied for P-FD towards the specific areas of interest since these were normalised for the total fixations duration. Mental and chronological age were standardised. An interaction term between the experimental group and the mental or chronological age was included in the model. Tuckey’s adjustment was considered to account for multiple comparisons. All tests were two-sided and statistical significance was set at 5%. All statistical analyses were performed with R^37^(4.0.2).

## Supplementary Information

Supplementary Table S1.

## Data Availability

The datasets used and analyzed during the current study are available from the corresponding author on reasonable request.

## Abbreviations

AS: Angelman syndrome
ASD: Autism spectrum disorder
ASG: Angelman syndrome group
CG: Control group
TF: Total fixations duration
P-FD: Proportion of fixations duration
