# Supplementary material for: Social attention and social-emotional modulation of attention in Angelman syndrome: an eye-tracking study
Source: Sci Rep. 2023 Feb 28;13:3375. doi: 10.1038/s41598-023-30199-6 (PMC9975183; doi:10.1038/s41598-023-30199-6)
Supplement: Supplementary file 1 — Supplementary Table S1. [file 41598_2023_30199_MOESM1_ESM.docx]

| **FIXATIONS DURATION** | **Videos** | **CG (N=21)**  **Mean (sd)** | **ASG (N=20)**  **Mean (sd)** | **Total (N=41)**  **Mean (sd)** | **p-value** |
| --- | --- | --- | --- | --- | --- |
| **Full screen** | **1** | 6836.2 (992.0) | 6526.1 (1913.6) | 6677.2 (1523.7) |  |
|  | **2** | 8306.8 (2141.3) | 6625.1 (2165.1) | 7444.4 (2289.3) |  |
|  | **3** | 8023.8 (1248.8) | 6584.4 (2537.2) | 7285.7 (2118.6) |  |
|  | **4** | 7285.6 (2112.4) | 5697.1 (2821.9) | 6449.5 (2604.9) |  |
|  | **5** | 6622.0 (1013.2) | 4708.4 (2646.7) | 5614.9 (2237.5) |  |
|  | **6** | 7655.9 (2148.8) | 5465.1 (2447.8) | 6502.9 (2535.2) |  |
|  | **7** | 6856.1 (2179.9) | 5565.9 (2191.4) | 6177.1 (2252.9) |  |
|  | **8** | 6622.4 (2155.2) | 5244.3 (3109.2) | 5914.8 (2741.2) |  |
|  | **TOTAL** | 7330.9 (1144.5) | 5806.0 (1591.3) | 6548.9 (1575.7) | 0.002 |
| **Areas of interest**  **(faces and actions)** | **1** | 4756.9 (1255.0) | 3794.2 (2100.5) | 4263.2 (1786.0) |  |
|  | **2** | 5338.5 (2070.3) | 3108.5 (2303.8) | 4194.9 (2441.1) |  |
|  | **3** | 5762.4 (1831.0) | 3763.2 (2260.8) | 4737.2 (2273.4) |  |
|  | **4** | 4439.4 (2588.8) | 2599.1 (2466.9) | 3470.8 (2659.2) |  |
|  | **5** | 4110.0 (1485.1) | 2309.1 (1952.8) | 3162.2 (1949.9) |  |
|  | **6** | 5216.3 (2182.4) | 2717.7 (1719.1) | 3901.2 (2303.1) |  |
|  | **7** | 4928.7 (2217.9) | 2825.2 (1986.6) | 3821.6 (2328.0) |  |
|  | **8** | 4419.9 (1959.5) | 2464.0 (2030.4) | 3415.5 (2203.8) |  |
|  | **TOTAL** | 4811.0 (1446.2) | 2947.7 (1542.8) | 3855.5 (1752.5) | <0.001 |
| **Actions** | **1** | 1531.9 (1016.2) | 2151.8 (1795.9) | 1849.8 (1483.4) |  |
|  | **2** | 1295.8 (1061.9) | 1189.2 (1140.2) | 1241.2 (1089.5) |  |
|  | **3** | 1745.4 (1451.3) | 1976.5 (1765.2) | 1863.9 (1603.0) |  |
|  | **4** | 735.2 (850.9) | 715.6 (960.5) | 724.9 (898.0) |  |
|  | **5** | 906.7 (1004.9) | 1004.5 (1213.4) | 958.2 (1105.7) |  |
|  | **6** | 1370.0 (1251.9) | 775.5 (1099.0) | 1057.1 (1196.2) |  |
|  | **7** | 1684.7 (1336.4) | 987.8 (995.5) | 1317.9 (1205.7) |  |
|  | **8** | 857.4 (795.7) | 685.6 (1110.2) | 769.2 (960.6) |  |
|  | **TOTAL** | 1273.3 (882.8) | 1184.7 (841.0) | 1227.9 (851.4) | 0.750 |
| **Faces** | **1** | 3225.0 (1233.1) | 1642.3 (1157.5) | 2413.4 (1425.6) |  |
|  | **2** | 4042.7 (1628.8) | 1919.2 (1759.2) | 2953.8 (1990.0) |  |
|  | **3** | 4017.1 (884.2) | 1786.8 (1146.3) | 2873.3 (1517.4) |  |
|  | **4** | 3704.2 (2366.2) | 1883.5 (1931.8) | 2745.9 (2310.4) |  |
|  | **5** | 3203.3 (984.8) | 1304.7 (1152.5) | 2204.0 (1432.0) |  |
|  | **6** | 3846.3 (1799.0) | 1942.2 (1333.7) | 2844.2 (1824.5) |  |
|  | **7** | 3244.1 (1251.0) | 1837.5 (1497.2) | 2503.8 (1541.6) |  |
|  | **8** | 3562.5 (1700.3) | 1778.4 (1752.8) | 2646.3 (1928.4) |  |
|  | **TOTAL** | 3537.7 (912.1) | 1763.0 (1079.2) | 2627.6 (1335.6) | <0.001 |

**Table 1S: Fixations duration (expressed in milliseconds) on of the full screen and the areas of interest (faces and actions) for each video**

| **NUMBER OF FIXATIONS** | **Videos** | **CG (N=21)**  **Mean (sd)** | **ASG (N=20)**  **Mean (sd)** | **Total (N=41)**  **Mean (sd)** | **p-value** |
| --- | --- | --- | --- | --- | --- |
| **Full screen time** | **1** | 19.6 (4.5) | 20.3 (6.7) | 19.9 (5.7) |  |
|  | **2** | 20.8 (6.8) | 20.4 (7.6) | 20.6 (7.1) |  |
|  | **3** | 19.1 (4.6) | 17.4 (5.8) | 18.2 (5.3) |  |
|  | **4** | 19.3 (7.4) | 19.4 (10.1) | 19.4 (8.8) |  |
|  | **5** | 20.3 (5.2) | 15.1 (8.3) | 17.5 (7.4) |  |
|  | **6** | 19.6 (5.1) | 17.4 (10.4) | 18.5 (8.3) |  |
|  | **7** | 18.4 (6.4) | 20.1 (12.7) | 19.3 (10.1) |  |
|  | **8** | 17.7 (4.5) | 16.4 (9.0) | 17.0 (7.1) |  |
|  | **TOTAL** | 19.6 (4.1) | 18.4 (6.7) | 19.0 (5.6) | 0.502 |
| **Areas of interest**  **(faces and actions)** | **1** | 12.5 (3.8) | 9.7 (5.3) | 11.1 (4.8) |  |
|  | **2** | 11.4 (4.0) | 7.8 (5.5) | 9.5 (5.1) |  |
|  | **3** | 11.9 (3.1) | 8.3 (4.3) | 10.1 (4.1) |  |
|  | **4** | 10.0 (5.4) | 7.3 (6.2) | 8.6 (5.9) |  |
|  | **5** | 11.2 (4.3) | 6.5 (5.1) | 8.7 (5.3) |  |
|  | **6** | 11.1 (3.9) | 7.1 (6.2) | 9.0 (5.5) |  |
|  | **7** | 11.5 (4.7) | 7.7 (7.6) | 9.5 (6.6) |  |
|  | **8** | 10.8 (3.6) | 5.8 (4.5) | 8.2 (4.7) |  |
|  | **TOTAL** | 11.2 (3.2) | 7.5 (4.2) | 9.3 (4.1) | 0.004 |
| **Actions** | **1** | 3.1 (2.5) | 3.7 (3.0) | 3.4 (2.7) |  |
|  | **2** | 2.1 (1.7) | 2.9 (3.1) | 2.5 (2.5) |  |
|  | **3** | 3.1 (2.0) | 3.3 (2.4) | 3.2 (2.2) |  |
|  | **4** | 1.7 (1.8) | 1.7 (1.7) | 1.7 (1.7) |  |
|  | **5** | 2.1 (2.2) | 1.8 (1.6) | 1.9 (1.9) |  |
|  | **6** | 2.4 (2.1) | 1.1 (1.4) | 1.7 (1.8) |  |
|  | **7** | 3.0 (2.2) | 2.4 (2.5) | 2.7 (2.3) |  |
|  | **8** | 1.8 (1.4) | 1.4 (2.2) | 1.6 (1.8) |  |
|  | **TOTAL** | 2.4 (1.5) | 2.3 (1.3) | 2.3 (1.4) | 0.755 |
| **Faces** | **1** | 9.4 (3.3) | 6.0 (3.8) | 7.6 (3.9) |  |
|  | **2** | 9.3 (3.3) | 4.9 (4.0) | 7.0 (4.3) |  |
|  | **3** | 8.9 (1.8) | 5.0 (2.9) | 6.9 (3.1) |  |
|  | **4** | 8.3 (4.1) | 5.6 (5.0) | 6.9 (4.8) |  |
|  | **5** | 9.1 (2.5) | 4.7 (4.7) | 6.8 (4.4) |  |
|  | **6** | 8.7 (3.1) | 6.1 (6.0) | 7.3 (5.0) |  |
|  | **7** | 8.5 (3.1) | 5.3 (5.7) | 6.8 (4.9) |  |
|  | **8** | 8.9 (3.1) | 4.3 (3.7) | 6.6 (4.1) |  |
|  | **TOTAL** | 8.8 (2.0) | 5.3 (3.7) | 7.0 (3.4) | <0.001 |
